# The peacock train does not handicap cursorial locomotor performance

**DOI:** 10.1038/srep36512

**Published:** 2016-11-02

**Authors:** Nathan K. Thavarajah, Peter G. Tickle, Robert L. Nudds, Jonathan R. Codd

**Affiliations:** 1 The Faculty of Biology, Medicine and Health, University of Manchester, Manchester, M13 9PT, UK; 2 School of Biomedical Science, University of Leeds, Leeds, LS2 9JT, UK

## Abstract

Exaggerated traits, like the peacock train, are recognized as classic examples of sexual selection. The evolution of sexual traits is often considered paradoxical as, although they enhance reproductive success, they are widely presumed to hinder movement and survival. Many exaggerated traits represent an additional mechanical load that must be carried by the animal and therefore may influence the metabolic cost of locomotion and constrain locomotor performance. Here we conducted respirometry experiments on peacocks and demonstrate that the exaggerated sexually selected train does not compromise locomotor performance in terms of the metabolic cost of locomotion and its kinematics. Indeed, peacocks with trains had a lower absolute and mass specific metabolic cost of locomotion. Our findings suggest that adaptations that mitigate any costs associated with exaggerated morphology are central in the evolution of sexually selected traits.

Charles Darwin first proposed that sexually selected characteristics were a direct result of inter- and/or intra-specific competition for mates during courtship^1^. Examples are widespread and include the elaborate breeding plumage of birds^2^, the courtship calls of invertebrates and anurans^3^,^4^, and the exaggerated structures that are exhibited by birds^5^, mammals^6^, invertebrates^7^ and fish^8^. Sexual selection, as it then became known, is one of the most important toolkits of natural selection. In terms of mate choice, sexually selected traits are those characteristics that have evolved for the sole purpose of attracting the opposite sex during courtship. In some rare cases it is the females that compete for males, but more common is the competition seen among the males to attract females. The most successful males being those that possess the most elaborate or exaggerated display characteristics^9^,^10^,^11^, resulting in selection pressures for secondary sexual trait elaboration^12^. Sexually selected traits, however, often appear cumbersome or conspicuous and are presumed to handicap the possessor in a number of ways, including their locomotor performance^13^. Understanding how animals move is important as all animals must expend energy to locomote in order to ensure predator avoidance, prey capture and reproduction, and they may only have limited reserves to do so^14^. Previous studies in birds have tended to focus on the influence of sexually selected traits during flight rather than terrestrial locomotion^5^,^15^,^16^,^17^,^18^,^19^. Yet, at the very least, exaggerated traits will add extra mass to a terrestrially locomoting animal which should require extra energy expenditure, as energy is needed to produce forces proportional to body mass (*M*_b_)^20^.

Peacocks (*Pavo cristatus*) are an iconic example of sexual selection and produce a spectacular train (up to 1.5 m in length) that is formed from their upper tail coverts prior to the breeding season. The peacock train is grown and discarded each year^10^ allowing investigation of the cost of trait possession under natural conditions. The ability to test the birds during the breeding season (when they have a train) and outside of the breeding season (once the train has been discarded) is important as this allows us to investigate if the birds are naturally compensating for the presence of the train in a way that artificially removing the train does not. During the breeding season, males establish and defend territories on the lek^21^. Here they display to females with an elaborate dance, erecting and fanning out their trains and produce a shimmering plumage display^22^. Females prefer to mate with peacocks that exhibit the most elaborate train^10^.

Zahavi^13^ saw the train as being cumbersome and specifically used the peacock as an example when describing the potential negative effects that may be imposed by a sexually selected trait on movement:

*“The excessive tail plumes of the peacock […] are obviously deleterious to the survival of the individual. […] The longer the plumes the more difficult it may be for the male to escape predators or to move about during everyday activity.” (Zahavi 1975, p211).*

However, there is a surprising lack of evidence to support the idea that locomotion in peacocks is in some way affected by sexually selected traits^19^. Accordingly, here we test the hypothesis that locomotor performance is hindered and the metabolic cost of locomotion is increased by the presence of the peacock train.

## Results

### Energetics

**Figure 1:**
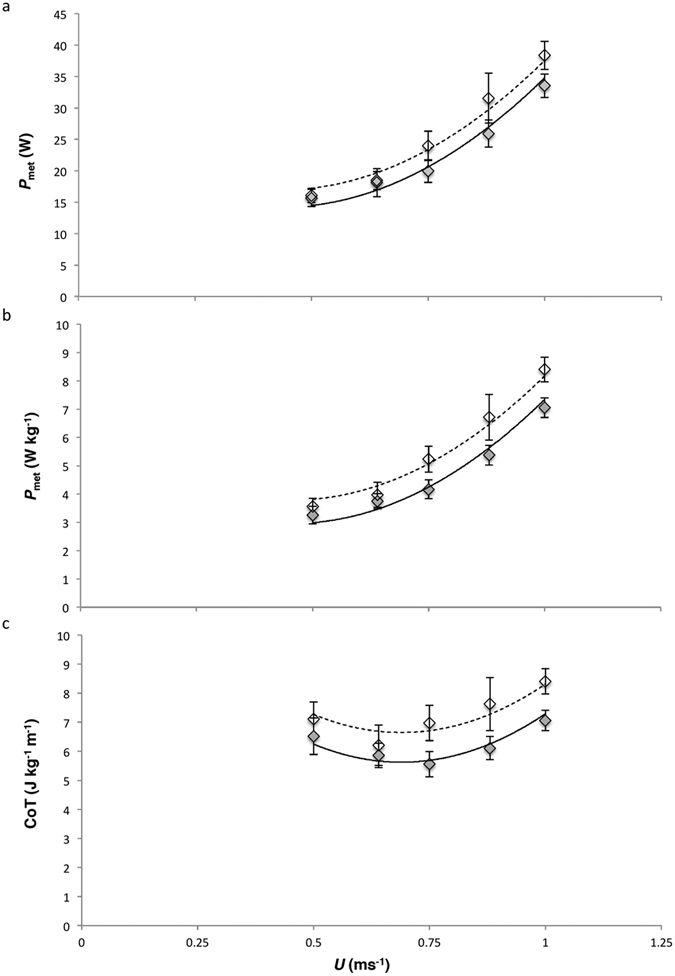
The energetic costs of walking for the peacocks during the breeding season with a full train (shaded symbols and solid lines) and outside the breeding season with a rudimentary train (open symbols and dashed lines). The lines of best fit are derived from the GLM model outputs and are defined by quadratic equations. (**a**) Absolute *P*_met_, (mean ± s.e.m) plotted against forward speed (*U*): full train, *P*_met_ = 26.31–55.79*U* + 64.28*U*^2^ and rudimentary train, *P*_met_ = 29.06–55.79*U* + 64.28*U*^2^. (**b**) Mass specific *P*_met_, (mean ± s.e.m) plotted against *U*: full train, *P*_met_ = 5.99–13.38*U* + 14.73*U*^2^; rudimentary train, *P*_met_ = 6.82–13.38*U* + 14.73*U*^2^. (**c**) CoT, (mean ± s.e.m) plotted against *U*: full train, CoT = 13.86–23.88*U* + 17.31*U*^2^; rudimentary train, CoT = 14.88–23.88*U* + 17.31*U*^2^. The original GLM model output for absolute *P*_met_ included a second explanatory variable, *M*_b_, therefore the lines of best fit describing the relationship between absolute *P*_met_ and *U* were derived by removing *M*_b_ from the model.

Absolute metabolic power consumption (*P*_met_) (W) increased curvilinearly with increasing speed (*U*) at a similar rate in each treatment and peacocks during the breeding season with fully grown trains had a lower absolute *P*_met_ during walking than outside of the breeding season (Table 1; Fig. 1a see supplementary information). Absolute *P*_met_ also increased with increasing *M*_b_ (Table 1). When energy expenditure was converted to mass-specific *P*_met_ (W kg^−1^), walking metabolic rate also increased curvilinearly at a similar rate with *U* for peacocks during both the breeding and non-breeding season. Peacocks had a lower mass-specific *P*_met_ across all speeds during the breeding season when the train was fully grown (Table 1; Fig. 1b). The relationship between net cost of transport (CoT_net_) and *U* was U-shaped and changed with *U* at a similar rate in both seasons. CoT_net_ was lower overall when the peacocks had their full trains, with the minimum CoT_net_ occurring at a higher *U*, than when the trains were in a rudimentary state (Table 1; Fig. 1c). There was no difference (*t*-test: *t* = 1.26, df = 17, *P* = 0.22) in absolute resting *P*_met_ (breeding season = 12.44 ± 0.58 W vs. non-breeding season = 13.49 ± 0.59 W) and no difference (*t*-test: *t* = 1.96, df = 17, *P* = 0.07) in mass specific resting *P*_met_ (breeding season = 2.62 ± 0.12 W vs. non-breeding season = 2.94 ± 0.12 W). RER values for all walking trials during both seasons were close to 1 (breeding: 1.01 ± 0.01; non-breeding: 0.95 ± 0.15).

### Kinematics

All of the kinematics parameters were influenced by *U*. DF, *t*_stance_ and *t*_swing_ all decreased with increasing *U*, whereas *l*_stride_ and *f*_stride_ increased with increasing *U*. However, there were no differences in any of the kinematics for peacocks in and out of the breeding season (Table 2, see supplementary information).

## Discussion

Contrary to our hypothesis, we found a lower metabolic cost of locomotion in peacocks during the breeding season when they had full trains. This lower cost is despite the fact the comparison was made between full and rudimentary trains. The lack of a negative effect of carrying the full train is surprising, not only because additional mass can elicit an increase in energy expenditure^20^, but also because we expect sexually selected traits to be burdensome during locomotion^13^.

Similar unexpected results were reported previously in another Galliforme species, the Svalbard rock ptarmigan (*Lagopus muta hyperborea*)^23^. The male Svalbard rock ptarmigan also exhibits a lower metabolic rate during locomotion during periods when *M*_b_ is increased, indicating that economical load carriage may be a more widespread phenomenon in Galliformes^24^. Furthermore, economical load carriage was also documented in other species that produce exaggerated sexually selected traits^25^, meaning economical carriage of exaggerated traits may also be widespread.

Several studies have made inferences about the costs of carrying exaggerated traits by either removing or artificially exaggerating them further^5^,^15^,^16^,^17^,^18^,^19^. However, while manipulating sexually selected traits by artificially removing them will produce clearly defined experimental testing of the role of the train it does mean any changes in general body condition, hormone levels, plumage quality or the influence of diurnal rhythms is not taken into account in the animals being investigated. Whilst it is not clear why having a train does not negatively affect peacock terrestrial locomotion energetics, it is possible that in the absence of any locomotor kinematics changes, it is driven by seasonal physiological and musculoskeletal changes. In other Galliformes, testosterone levels are related to the onset of sexually selected ornamental and behavioural displays^26^,^27^. An increase in testosterone could stimulate muscle growth in peacocks^28^,^29^, which could in turn influence seasonal differences in locomotion^30^,^31^,^32^.

Recently, Askew^19^ found that the train did not reduce take off flight performance, which combined with our results, indicates that neither terrestrial or volant locomotion are negatively impacted by the exaggerated sexually selected train of peacocks. The maintenance of economical locomotion during periods when locomotor behaviour is focused on procuring a mate is likely to be particularly important for reducing the overall costs^13^. The metabolic costs associated with locomotion are a key component of the daily energy expenditure of many species of bird such as the peacock. Territorial behaviour is a key component of mating success in peacocks and during this time intra-sexual agonistic behaviour is frequent among the birds^21^. Therefore, we suggest that the lower CoT when the train is fully expressed is an adaptation that enables peacocks to balance energy requirements during periods of high-energy use and low food acquisition such as territorial behavior and displays used to attract mates^33^.

Interestingly, for peacocks, the relationship between metabolic power consumption (*P*_met_) and speed (*U*) for walking during both experimental periods was curvilinear, which is also seen in other animals including some species of large bird^34^,^35^,^36^,^37^,^38^ but does deviate from the more common linear relationship found in many species^39^,^40^. As a result the cost of transport (CoT) (J kg^−1^ m^−1^) curve was U-shaped, indicating that peacocks would benefit from selecting an intermediate walking speed of 0.75 ms^−1^ which represents their minimum CoT over the speed range we investigated. A U-shaped curve during walking locomotion has also been observed in another large cursorial bird, the emu (*Dromaius novaehollandiae*), and field observations revealed that the self-selected speeds preferred by emu were tightly clustered around the lowest point of the curve in line with predictions they chose this speed to minimize the CoT^34^. It would interesting to know if peacocks’ preferred speeds in the wild would similarly correspond to the speeds matched to the minimum CoT in this study.

As is to be expected there will be a trade off between natural and sexual selection of traits. While sexual selection may act to enhance trait size natural selection will minimize the effect of this on for example predation risk. This trade off means that when traits are exhibited that appear cumbersome, natural selection may select for co-adaptations for the mitigation of any additional costs^41^,^42^. It is important to stress that our results focus entirely on the costs associated with locomotion, there are of course numerous other costs related to sexual selection that may be incurred, indeed for peacocks these may lie in the development of the trait and in its display^43^,^44^ rather than the inherent cost of moving around with the trait.

## Methods

### Animals

We conducted experiments on adult peacocks in the breeding season (May) (n = 10, *M*_b_: 4.76 ± 0.10 kg) when the train was fully-grown (1.5 m) and once breeding had been completed (November) (n = 9, 4.58 ± 0.09 kg) when the train was in a rudimentary state of growth. The experimental time periods were chosen to allow for natural comparison of the effects of carrying the train (i.e. in birds with and without their trains). However, after feather molt, birds are often very stressed^32^ meaning the peacocks were reluctant to move on the treadmill once the train was completely molted. Consequently, walking data could not be collected until the train was in a state of rudimentary regrowth not extending beyond the body of the bird. The different sample sizes are a result of one male damaging his leg at the farm, where the peacocks were housed between the experimental time periods, and this bird was therefore unavailable during the non-breeding season trials. Experimental procedures were carried out under ethical approval from the University of Manchester Ethics Committee and in accordance with the Animals (Scientific Procedures) Act 1986, covered by a UK Home Office project licence (40/3549) held by Dr Codd.

### Respirometry

Open flow indirect calorimetry was used to measure oxygen consumption (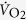) and carbon dioxide (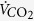) production (all equipment and computer programs Sable Systems International^®^, Las Vegas, U.S.A.). Trial were conducted inside a Perspex^**©**^ box (volume 620 L) mounted on a treadmill (Professional Model, Fit Fur Life, Surrey, UK). The box was designed so that the rear panel was sloping to both minimize the volume of the box and to ensure that the train did not touch the box itself at any point. Air was pulled through using a Flow-Kit 2000, at 450 L min^−1^ (FR). The excurrent flow was then subsampled at 0.1 L min^−1^ for gas analysis. Water vapour pressure (WVP) was recorded using an RH-300. Water was then scrubbed using calcium chloride (2–6 mm granular, Merck, Darmstadt, Germany). The sample was then drawn through CA-10 carbon dioxide analyser before CO_2_ was scrubbed using soda lime (2–5 mm granular, Sigma Aldrich, Steinheim, Germany) and finally O_2_ concentration and barometric pressure (BP) were measured using an Oxilla II. Ambient air (scrubbed of H_2_O and CO_2_ as before) was simultaneously drawn through the second channel of the Oxilla II at 0.1 L min-1 by a separate pump (SS-3) to enable calculation of differential O_2_ concentration (ΔO_2_). Background CO_2_ was subtracted from the measurements to calculate differential CO_2_ concentration (ΔCO_2_). Outputs were recorded using a UI-2 and analysed using ExpeData Software. The accuracy of the system (± 4%) was validated by N_2_ dilution tests^45^. Primary flow rates were adjusted to dry-corrected flow rates (FR_c_) to account for the H_2_0 scrubbed from the air samples prior to gas measurement using equation 1: (all equations from^46^):


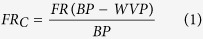



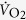
 was calculated using


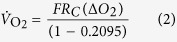


and 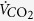 using


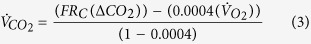


Metabolic power consumption (*P*_met_, W or W kg^−1^) and cost of transport (CoT) (J kg^−1^ m^−1^) was converted from 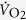, using the respiratory exchange ratio (RER: 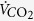: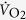) and thermal equivalents taken from^47^. All energetics data was analyzed as net-*P*_met_ or net-CoT by subtracting resting metabolic rate (RMR) from locomotor metabolic rate (both from the same trial). RER’s were monitored for all trials to ensure that the birds were locomoting aerobically^39^.

Prior to the experimental trials the birds were trained to walk inside the Perspex box at randomized speeds. The birds were exercised on the treadmill at five speeds (Fig. 1, 0.5, 0.64, 0.75, 0.88, 1 ms^−1^). During the breeding season experiments, 7 birds with full trains walked at 0.5, 0.64, and 0.88 ms^−1^, 8 at 0.75 ms^−1^ and 5 at 1 ms^−1^. During the non-breeding season experiments, 7 birds with rudimentary trains walked at 0.5, 0.64, and 1 ms^−1^, 8 at 0.75 ms^−1^ and 5 at 0.75 ms^−1^. The speeds were randomized with a maximum of 4 speeds per trial. The protocol for all experiments was identical: after entering the respirometry chamber the birds were given a warm up trial at the lowest speed and were then rested for 5 minutes before being walked for data collection. The duration of walking at each speed was 5–6 minutes, or until the birds reached steady state metabolism, the final minute of the plateau was used for data analyses. Each walking trial was followed by a resting period of 5 minutes before the peacocks were walked at the next speed. The birds were rested for at least 1 day between trials.

### Kinematics

The peacocks were filmed in lateral view using a Sony Handycam (HDR cx410VE, Sony, UK) at either 25 Hz. (≤0.75 ms^−1^) or 100 Hz (≥0.75 ms^−1^). Footfall of the left foot was tracked (Tracker software v. 4.84 Open Source Physics) to quantify duty factor (DF), stride frequency (*f*_stride_) and stride length (*l*_stride_), stance time (*t*_stance_) and swing time (*t*_swing_).

### Statistical Analyses

Statistical analyses were performed in R 2.14.0 GUI 1.42 Leopard build 64-bit^48^. Plot visualization showed the energetics data to be curvilinear, with and without transformation of the independent variables. Therefore differences in net *P*_met_ (W and W kg^−1^) and CoT, of peacocks between seasons were tested using a quadratic general linear model (GLM). All of the kinematics parameters (DF, *f*_stride_, *l*_stride_, *t*_stance_ and *t*_swing_) were analyzed using standard ANCOVA. Shapiro–Wilk tests were performed on the standardised residuals generated by each statistical model to ensure that the data conformed to a normal distribution. The statistical results were derived from the minimum adequate model i.e. non-significant interaction terms were stepwise deleted from the model. Resting metabolic rate and *M*_b_ of individuals with full and rudimentary trains were compared using a students *t*-test. Data sets supporting this article are included in the electronic supplementary material, ESM:1.

## Additional Information

**How to cite this article**: Thavarajah, N. K. *et al.* The peacock train does not handicap cursorial locomotor performance. *Sci. Rep.* **6**, 36512; doi: 10.1038/srep36512 (2016).

**Publisher’s note**: Springer Nature remains neutral with regard to jurisdictional claims in published maps and institutional affiliations.

## Supplementary Material

Supplementary Information

## Figures and Tables

**Table 1:** Summary of the quadratic GLM models for the energetics of walking. Both the final statistical model and the non-significant interaction terms, which were removed by stepwise deletion, are shown.

Parameter	Non significant interaction terms	Final GLM
Absolute *P*_met_ (W)	*U*^x^season^x^*M*_b_ (*F*_1,59_ = 0.15, *P* = 0.70)	*M*_b_ (*F*_1,63_ = 16.46, *P* < 0.001)
	season^x^*M*_b_ (*F*_1,60_ = 0.53, *P* = 0.47)	*U* (*F*_1,63_ = 9.55, *P* < 0.01)
	*U*^x^*M*_b_ (*F*_1,61_ = 1.61, *P* = 0.21)	season (*F*_1,63_ = 5.68, *P* < 0.05)
	*U*^x^season (*F*_1,62_ = 1.61, *P* = 0.21)	
Mass specific *P*_met_ (W kg^−1^)	*U*^x^season (*F*_1,63_ = 3.08, *P* = 0.08)	*U* (*F*_1,64_=10.73, *P* < 0.01)
		season (*F*_1,64_ = 10.72, *P* < 0.01)
CoT (J kg^−1^ m^−1^)	*U*^x^season (*F*_1,63_ = 1.04, *P* = 0.31)	*U* (*F*_1,64_ = 8.02, *P* < 0.01)
		season (*F*_1,64_ = 17.70, *P* < 0.01)

**Table 2:** Summary of the ANCOVA models for the kinematics parameters.

Parameter	Non significant interaction terms	Final ANCOVA	Coefficients (from model output)
DF	*U*^x^season (*F*_1,64_ = 0.41, *P* = 0.52)	*U* (*F*_1,65_ = 20.75, *P* < 0.001)	Breeding season = 0.72 + −0.08*U*
		season (*F*_1,65_ = 0.003, *P* = 0.95)	Non-breeding season = 0.72 + −0.08*U*
*f*_stride_	*U*^x^season (*F*_1,64_ = 0.97, P = 0.33)	*U* (*F*_1,65_ = 531.21, *P* < 0.001)	Breeding season = 0.38 + 0.99*U*
		season (*F*_1,65_ = 0.03, *P* = 0.87)	Non-breeding season = 0.38 + 0.99*U*
*l*_stride_	*U*^x^season (*F*_1,64_ = 0.03, *P* = 0.85)	*U* (*F*_1,65_ = 93.77, *P* < 0.001)	Breeding season = 0.45 + 0.27*U*
		season (*F*_1,65_ = 0.35, *P* = 0.56)	Non-breeding season = 0.45 + 0.27*U*
*t*_stance_	*U*^x^season (*F*_1,64_ = 2.33, *P* = 0.13)	*U* (*F*_1,65_ = 413.89, *P* < 0.001)	Breeding season = 1.07 + −0.63*U*
		season (_*F*1,65_ = 2.19, *P* = 0.14)	Non-breeding season = 1.08 + −0.63*U*
*t*_swing_	*U*^x^season (*F*_1,64_ = 0.001, *P* = 0.97)	*U* (*F*_1,65_ = 71.04, *P* < 0.001)	Breeding season = 0.45 + −0.20*U*
		season (*F*_1,65_ = 0.08, *P* = 0.78)	Non-breeding season = 0.45 + −0.20*U*
