# Supplementary material for: The peacock train does not handicap cursorial locomotor performance
Source: Sci Rep. 2016 Nov 2;6:36512. doi: 10.1038/srep36512 (PMC5090354; doi:10.1038/srep36512)
Supplement: Supplementary Information [file srep36512-s1.doc]

The peacock train does not handicap cursorial locomotor performance

Nathan K Thavarajah^1^, Peter G Tickle^2^, Robert L Nudds^1^ and Jonathan R Codd^1^*

^1^Faculty of Life Sciences, University of Manchester, Manchester, M13 9PT, UK, ^2^School of Biomedical Science, University of Leeds, Leeds, LS2 9JT, UK.

Electronic Supplementary Material: respirometry raw data

*Corresponding author

Jonathan R Codd

FLS, University of Manchester

Michael Smith Bldg

Oxford Rd

Manchester

M139PT

UK

Tel: +44(0) 161 275 5474

Email: jonathan.codd@manchester.ac.uk

Key words: exaggerated traits, energy expenditure, cost of transport

**Running Title: costs of sexual selection**

***Supplementary Table 1: Energetics raw data***

| Date | bird | mass | *U* | MFR | BP | WVP | SEASON | DRY_MFR | DRY_VO2 | DRY_VCO2 | RQ | RQ_CAL_FACTOR | CAL/MIN | J/S | J/S_net |
| --- | --- | --- | --- | --- | --- | --- | --- | --- | --- | --- | --- | --- | --- | --- | --- |
| 28/4/14 | M1 | 4.2 | 0 | 450 | 92.15 | 1.11 | SUMMER | 444.58 | 40.50 | 44.41 | 1.10 | 5.16 | 209.11 | 14.58 | 14.58 |
| 2/5/14 | M1 | 4.15 | 0 | 450 | 93.43 | 0.88 | SUMMER | 445.76 | 24.63 | 19.90 | 0.81 | 4.81 | 118.44 | 8.26 | 8.26 |
| 7/5/15 | M10 | 4.7 | 0 | 450 | 92.4 | 0.91 | SUMMER | 445.57 | 42.19 | 46.33 | 1.10 | 5.17 | 217.93 | 15.20 | 15.20 |
| 9/5/15 | M10 | 4.55 | 0 | 450 | 91.98 | 0.98 | SUMMER | 445.21 | 22.80 | 25.75 | 1.13 | 5.20 | 118.67 | 8.28 | 8.28 |
| 11/5/15 | M10 | 4.6 | 0 | 450 | 92.3 | 1.03 | SUMMER | 444.98 | 34.88 | 32.62 | 0.94 | 4.97 | 173.18 | 12.08 | 12.08 |
| 5/5/15 | M11 | 5 | 0 | 450 | 89.81 | 1.37 | SUMMER | 443.14 | 40.02 | 41.69 | 1.04 | 5.10 | 203.96 | 14.22 | 14.22 |
| 7/5/15 | M11 | 4.9 | 0 | 450 | 92.38 | 0.98 | SUMMER | 445.23 | 42.47 | 43.34 | 1.02 | 5.07 | 215.34 | 15.02 | 15.02 |
| 9/5/15 | M11 | 4.9 | 0 | 450 | 92.07 | 1 | SUMMER | 445.11 | 33.23 | 29.52 | 0.89 | 4.91 | 163.07 | 11.37 | 11.37 |
| 11/5/15 | M11 | 4.9 | 0 | 450 | 92.28 | 1.01 | SUMMER | 445.07 | 26.14 | 27.82 | 1.06 | 5.12 | 133.94 | 9.34 | 9.34 |
| 5/5/15 | M12 | 5.05 | 0 | 450 | 90.15 | 1.01 | SUMMER | 444.96 | 36.27 | 35.78 | 0.99 | 5.03 | 182.38 | 12.72 | 12.72 |
| 9/5/15 | M12 | 5 | 0 | 450 | 92.35 | 1.01 | SUMMER | 445.08 | 28.24 | 31.00 | 1.10 | 5.17 | 145.85 | 10.17 | 10.17 |
| 11/5/15 | M12 | 4.9 | 0 | 450 | 92.29 | 1.36 | SUMMER | 443.37 | 35.88 | 29.57 | 0.82 | 4.83 | 173.28 | 12.08 | 12.08 |
| 28/4/14 | M3 | 5.1 | 0 | 450 | 92.17 | 1.14 | SUMMER | 444.43 | 37.54 | 41.78 | 1.11 | 5.18 | 194.59 | 13.57 | 13.57 |
| 30/4/14 | M3 | 5.1 | 0 | 450 | 92.14 | 1.28 | SUMMER | 443.75 | 40.82 | 46.71 | 1.14 | 5.22 | 213.19 | 14.87 | 14.87 |
| 2/5/14 | M3 | 5 | 0 | 450 | 93.5 | 0.85 | SUMMER | 445.91 | 30.40 | 29.14 | 0.96 | 4.99 | 151.83 | 10.59 | 10.59 |
| 28/4/14 | M4 | 4.55 | 0 | 450 | 92.17 | 1.12 | SUMMER | 444.53 | 25.87 | 25.85 | 1.00 | 5.04 | 130.50 | 9.10 | 9.10 |
| 30/4/14 | M4 | 4.55 | 0 | 450 | 92.09 | 1.2 | SUMMER | 444.14 | 28.36 | 30.71 | 1.08 | 5.15 | 145.95 | 10.18 | 10.18 |
| 2/5/14 | M4 | 4.55 | 0 | 450 | 93.45 | 0.85 | SUMMER | 445.91 | 22.18 | 19.97 | 0.90 | 4.92 | 109.17 | 7.61 | 7.61 |
| 25/4/14 | M5 | 4.5 | 0 | 450 | 92.75 | 1.22 | SUMMER | 444.08 | 27.67 | 31.29 | 1.13 | 5.21 | 144.05 | 10.04 | 10.04 |
| 28/4/14 | M5 | 4.45 | 0 | 450 | 92.16 | 1.13 | SUMMER | 444.48 | 38.36 | 43.62 | 1.14 | 5.21 | 200.00 | 13.95 | 13.95 |
| 30/4/14 | M5 | 4.5 | 0 | 450 | 92.15 | 1.25 | SUMMER | 443.90 | 34.34 | 38.58 | 1.12 | 5.20 | 178.44 | 12.44 | 12.44 |
| 25/4/14 | M6 | 5.1 | 0 | 450 | 92.75 | 1.24 | SUMMER | 443.98 | 40.27 | 45.83 | 1.14 | 5.21 | 210.00 | 14.64 | 14.64 |
| 30/4/14 | M6 | 5 | 0 | 450 | 92.11 | 1.22 | SUMMER | 444.04 | 48.16 | 48.38 | 1.00 | 5.05 | 243.25 | 16.96 | 16.96 |
| 2/5/14 | M6 | 4.95 | 0 | 450 | 93.4 | 0.91 | SUMMER | 445.62 | 29.98 | 23.51 | 0.78 | 4.78 | 143.28 | 9.99 | 9.99 |
| 30/5/14 | M7 | 4.7 | 0 | 450 | 92.06 | 1.19 | SUMMER | 444.18 | 48.05 | 39.36 | 0.82 | 4.82 | 231.75 | 16.16 | 16.16 |
| 5/5/15 | M8 | 5.1 | 0 | 450 | 90.01 | 1.1 | SUMMER | 444.50 | 49.80 | 50.57 | 1.02 | 5.06 | 252.21 | 17.59 | 17.59 |
| 7/5/15 | M8 | 5.1 | 0 | 450 | 92.36 | 1.04 | SUMMER | 444.93 | 27.63 | 28.61 | 1.04 | 5.09 | 140.58 | 9.80 | 9.80 |
| 9/5/15 | M8 | 5.05 | 0 | 450 | 92.22 | 1.02 | SUMMER | 445.02 | 38.30 | 29.64 | 0.77 | 4.77 | 182.59 | 12.73 | 12.73 |
| 11/5/15 | M8 | 5.05 | 0 | 450 | 92.29 | 0.96 | SUMMER | 445.32 | 31.51 | 33.82 | 1.07 | 5.14 | 161.81 | 11.28 | 11.28 |
| 7/5/15 | M10 | 4.7 | 0.5 | 450 | 92.4 | 0.93 | SUMMER | 445.47 | 92.21 | 92.04 | 1.00 | 5.04 | 465.01 | 32.43 | 17.23 |
| 11/5/15 | M10 | 4.6 | 0.5 | 450 | 92.32 | 1.03 | SUMMER | 444.98 | 92.07 | 83.61 | 0.91 | 4.93 | 454.10 | 31.67 | 19.59 |
| 5/5/15 | M11 | 5 | 0.5 | 450 | 89.69 | 1.41 | SUMMER | 442.93 | 98.47 | 112.63 | 1.14 | 5.22 | 514.16 | 35.85 | 21.63 |
| 11/5/15 | M11 | 4.9 | 0.5 | 450 | 92.27 | 1.01 | SUMMER | 445.07 | 61.62 | 65.27 | 1.06 | 5.12 | 315.34 | 21.99 | 12.65 |
| 5/5/15 | M12 | 5.05 | 0.5 | 450 | 90.15 | 1.01 | SUMMER | 444.96 | 80.59 | 78.36 | 0.97 | 5.01 | 403.83 | 28.16 | 15.44 |
| 9/5/15 | M12 | 5 | 0.5 | 450 | 92.3 | 1.02 | SUMMER | 445.03 | 77.42 | 76.02 | 0.98 | 5.02 | 388.88 | 27.12 | 16.95 |
| 28/4/14 | M4 | 4.55 | 0.5 | 450 | 92.17 | 1.14 | SUMMER | 444.43 | 67.60 | 73.38 | 1.09 | 5.15 | 348.15 | 24.28 | 15.18 |
| 30/4/14 | M6 | 5 | 0.5 | 450 | 92.11 | 1.23 | SUMMER | 443.99 | 71.19 | 72.48 | 1.02 | 5.07 | 360.72 | 25.15 | 8.19 |
| 30/5/14 | M7 | 4.7 | 0.5 | 450 | 92.07 | 1.21 | SUMMER | 444.09 | 106.02 | 89.09 | 0.84 | 4.85 | 514.08 | 35.85 | 19.69 |
| 5/5/15 | M8 | 5.1 | 0.5 | 450 | 89.98 | 1.08 | SUMMER | 444.60 | 77.25 | 87.02 | 1.13 | 5.20 | 401.73 | 28.01 | 10.43 |
| 9/5/15 | M8 | 5.05 | 0.5 | 450 | 92.18 | 1.01 | SUMMER | 445.07 | 93.17 | 88.98 | 0.96 | 4.99 | 464.89 | 32.42 | 19.69 |
| 9/5/15 | M10 | 4.55 | 0.64 | 450 | 91.98 | 1.03 | SUMMER | 444.96 | 90.19 | 93.35 | 1.04 | 5.09 | 458.89 | 32.00 | 23.72 |
| 11/5/15 | M10 | 4.6 | 0.64 | 450 | 92.31 | 1.02 | SUMMER | 445.03 | 80.21 | 74.69 | 0.93 | 4.96 | 397.89 | 27.75 | 15.67 |
| 5/5/15 | M11 | 5 | 0.64 | 450 | 89.73 | 1.41 | SUMMER | 442.93 | 87.04 | 85.55 | 0.98 | 5.02 | 437.29 | 30.49 | 16.27 |
| 9/5/15 | M11 | 4.9 | 0.64 | 450 | 92.04 | 1 | SUMMER | 445.11 | 95.54 | 90.69 | 0.95 | 4.98 | 476.05 | 33.20 | 21.83 |
| 11/5/15 | M12 | 4.9 | 0.64 | 450 | 92.29 | 1.37 | SUMMER | 443.32 | 91.84 | 75.41 | 0.82 | 4.83 | 443.15 | 30.90 | 18.82 |
| 28/4/14 | M3 | 5.1 | 0.64 | 450 | 92.16 | 1.13 | SUMMER | 444.48 | 79.15 | 90.91 | 1.15 | 5.23 | 413.75 | 28.85 | 15.28 |
| 2/5/14 | M3 | 5 | 0.64 | 450 | 93.5 | 0.87 | SUMMER | 445.81 | 83.56 | 94.63 | 1.13 | 5.21 | 435.14 | 30.34 | 19.76 |
| 28/4/14 | M4 | 4.55 | 0.64 | 450 | 92.17 | 1.13 | SUMMER | 444.48 | 55.78 | 56.62 | 1.02 | 5.06 | 282.43 | 19.69 | 10.59 |
| 2/5/14 | M4 | 4.55 | 0.64 | 450 | 93.44 | 0.86 | SUMMER | 445.86 | 50.52 | 56.92 | 1.13 | 5.20 | 262.74 | 18.32 | 10.71 |
| 2/5/14 | M6 | 4.95 | 0.64 | 450 | 93.4 | 0.95 | SUMMER | 445.42 | 88.55 | 82.84 | 0.94 | 4.97 | 439.72 | 30.66 | 20.67 |
| 7/5/15 | M8 | 5.1 | 0.64 | 450 | 92.36 | 1.04 | SUMMER | 444.93 | 96.02 | 90.47 | 0.94 | 4.97 | 477.59 | 33.30 | 23.50 |
| 11/5/15 | M8 | 5.05 | 0.64 | 450 | 92.29 | 0.99 | SUMMER | 445.17 | 95.06 | 91.61 | 0.96 | 5.00 | 475.33 | 33.15 | 21.86 |
| 2/5/14 | M1 | 4.15 | 0.75 | 450 | 93.4 | 0.89 | SUMMER | 445.71 | 63.24 | 71.70 | 1.13 | 5.21 | 329.44 | 22.97 | 14.71 |
| 9/5/15 | M10 | 4.55 | 0.75 | 450 | 91.98 | 1.02 | SUMMER | 445.01 | 88.37 | 91.04 | 1.03 | 5.08 | 449.10 | 31.32 | 23.04 |
| 11/5/15 | M10 | 4.6 | 0.75 | 450 | 92.31 | 1.07 | SUMMER | 444.78 | 96.75 | 97.13 | 1.00 | 5.05 | 488.55 | 34.07 | 21.99 |
| 7/5/15 | M11 | 4.9 | 0.75 | 450 | 92.38 | 0.94 | SUMMER | 445.42 | 105.39 | 103.43 | 0.98 | 5.02 | 529.27 | 36.91 | 21.89 |
| 9/5/15 | M11 | 4.9 | 0.75 | 450 | 92.07 | 1 | SUMMER | 445.11 | 100.99 | 103.36 | 1.02 | 5.07 | 512.42 | 35.73 | 24.36 |
| 5/5/15 | M12 | 5.05 | 0.75 | 450 | 90.11 | 1 | SUMMER | 445.01 | 100.44 | 111.68 | 1.11 | 5.18 | 520.53 | 36.30 | 23.58 |
| 11/5/15 | M12 | 4.9 | 0.75 | 450 | 92.29 | 1.34 | SUMMER | 443.47 | 92.01 | 86.97 | 0.95 | 4.98 | 458.02 | 31.94 | 19.86 |
| 28/4/14 | M3 | 5.1 | 0.75 | 450 | 92.17 | 1.18 | SUMMER | 444.24 | 77.88 | 84.69 | 1.09 | 5.15 | 401.27 | 27.98 | 14.41 |
| 2/5/14 | M3 | 5 | 0.75 | 450 | 93.5 | 0.87 | SUMMER | 445.81 | 81.38 | 88.60 | 1.09 | 5.15 | 419.41 | 29.25 | 18.66 |
| 28/4/14 | M4 | 4.55 | 0.75 | 450 | 92.17 | 1.13 | SUMMER | 444.48 | 59.93 | 60.82 | 1.01 | 5.06 | 303.42 | 21.16 | 12.06 |
| 30/5/14 | M7 | 4.7 | 0.75 | 450 | 92.07 | 1.21 | SUMMER | 444.09 | 107.73 | 104.20 | 0.97 | 5.00 | 539.15 | 37.60 | 21.44 |
| 7/5/15 | M8 | 5.1 | 0.75 | 450 | 92.36 | 1.04 | SUMMER | 444.93 | 106.59 | 103.81 | 0.97 | 5.01 | 534.32 | 37.26 | 27.46 |
| 9/5/15 | M8 | 5.05 | 0.75 | 450 | 92.16 | 1.01 | SUMMER | 445.07 | 117.41 | 107.30 | 0.91 | 4.94 | 579.92 | 40.44 | 27.71 |
| 2/5/14 | M1 | 4.15 | 0.88 | 450 | 93.43 | 0.89 | SUMMER | 445.71 | 72.13 | 82.94 | 1.15 | 5.23 | 377.16 | 26.30 | 18.04 |
| 7/5/15 | M10 | 4.7 | 0.88 | 450 | 92.4 | 0.93 | SUMMER | 445.47 | 104.10 | 109.69 | 1.05 | 5.11 | 532.06 | 37.10 | 21.91 |
| 7/5/15 | M11 | 4.9 | 0.88 | 450 | 92.38 | 0.98 | SUMMER | 445.23 | 132.22 | 132.47 | 1.00 | 5.05 | 667.37 | 46.54 | 31.52 |
| 11/5/15 | M11 | 4.9 | 0.88 | 450 | 92.29 | 1.01 | SUMMER | 445.08 | 132.04 | 128.14 | 0.97 | 5.01 | 661.35 | 46.12 | 36.78 |
| 9/5/15 | M12 | 5 | 0.88 | 450 | 92.32 | 1.03 | SUMMER | 444.98 | 115.90 | 111.69 | 0.96 | 5.00 | 579.55 | 40.41 | 30.24 |
| 2/5/14 | M6 | 4.95 | 0.88 | 450 | 93.4 | 0.95 | SUMMER | 445.42 | 99.17 | 111.39 | 1.12 | 5.20 | 515.32 | 35.93 | 25.94 |
| 30/5/14 | M7 | 4.7 | 0.88 | 450 | 92.06 | 1.23 | SUMMER | 443.99 | 111.28 | 95.36 | 0.86 | 4.87 | 541.87 | 37.79 | 21.63 |
| 5/5/15 | M8 | 5.1 | 0.88 | 450 | 89.91 | 1.16 | SUMMER | 444.19 | 129.62 | 146.84 | 1.13 | 5.21 | 675.06 | 47.07 | 29.49 |
| 9/5/15 | M8 | 5.05 | 0.88 | 450 | 92.2 | 1.04 | SUMMER | 444.92 | 124.71 | 106.27 | 0.85 | 4.86 | 606.50 | 42.29 | 29.56 |
| 2/5/14 | M1 | 4.15 | 1 | 450 | 93.4 | 0.9 | SUMMER | 445.66 | 99.70 | 115.33 | 1.16 | 5.24 | 522.18 | 36.41 | 28.15 |
| 11/5/15 | M10 | 4.6 | 1 | 450 | 92.3 | 1.03 | SUMMER | 444.98 | 139.89 | 140.98 | 1.01 | 5.05 | 707.09 | 49.31 | 37.23 |
| 5/5/15 | M11 | 5 | 1 | 450 | 89.66 | 1.4 | SUMMER | 442.97 | 140.66 | 148.02 | 1.05 | 5.11 | 718.67 | 50.11 | 35.89 |
| 9/5/15 | M11 | 4.9 | 1 | 450 | 92.04 | 1.01 | SUMMER | 445.06 | 140.31 | 143.73 | 1.02 | 5.07 | 712.07 | 49.66 | 38.28 |
| 5/5/15 | M12 | 5.05 | 1 | 450 | 90.14 | 1.01 | SUMMER | 444.96 | 122.97 | 134.68 | 1.10 | 5.16 | 634.76 | 44.26 | 31.55 |
| 9/5/15 | M12 | 5 | 1 | 450 | 92.29 | 1.04 | SUMMER | 444.93 | 107.31 | 117.17 | 1.09 | 5.16 | 553.48 | 38.60 | 28.43 |
| 5/5/15 | M8 | 5.1 | 1 | 450 | 89.93 | 1.11 | SUMMER | 444.45 | 139.20 | 155.99 | 1.12 | 5.19 | 722.88 | 50.41 | 32.82 |
| 11/5/15 | M8 | 5.05 | 1 | 450 | 92.29 | 0.98 | SUMMER | 445.22 | 139.06 | 136.96 | 0.98 | 5.03 | 698.97 | 48.74 | 37.46 |
| 18/11/13 | M1 | 4.2 | 0 | 450 | 94.18 | 1.07 | WINTER | 444.89 | 41.49 | 35.41 | 0.85 | 4.87 | 201.87 | 14.08 | 14.08 |
| 2/12/13 | M1 | 4 | 0 | 450 | 94.18 | 0.92 | WINTER | 445.60 | 39.60 | 37.76 | 0.95 | 4.99 | 197.52 | 13.77 | 13.77 |
| 4/12/13 | M1 | 4.2 | 0 | 450 | 93.75 | 0.88 | WINTER | 445.78 | 20.00 | 18.37 | 0.92 | 4.94 | 98.90 | 6.90 | 6.90 |
| 6/12/13 | M1 | 3.9 | 0 | 450 | 93.56 | 0.84 | WINTER | 445.96 | 34.75 | 29.69 | 0.85 | 4.87 | 169.08 | 11.79 | 11.79 |
| 16/11/15 | M10 | 4.65 | 0 | 450 | 91.63 | 0.98 | WINTER | 445.19 | 35.62 | 31.71 | 0.89 | 4.91 | 174.92 | 12.20 | 12.20 |
| 20/11/15 | M10 | 4.6 | 0 | 450 | 91.37 | 1.11 | WINTER | 444.53 | 28.81 | 26.73 | 0.93 | 4.96 | 142.78 | 9.96 | 9.96 |
| 22/11/15 | M10 | 4.6 | 0 | 450 | 92.64 | 0.91 | WINTER | 445.58 | 31.50 | 35.28 | 1.12 | 5.19 | 163.55 | 11.40 | 11.40 |
| 16/11/15 | M11 | 4.7 | 0 | 450 | 91.65 | 0.98 | WINTER | 445.19 | 32.22 | 33.82 | 1.05 | 5.11 | 164.53 | 11.47 | 11.47 |
| 18/11/15 | M11 | 4.75 | 0 | 450 | 91.04 | 1.27 | WINTER | 443.72 | 31.72 | 23.24 | 0.73 | 4.72 | 149.62 | 10.43 | 10.43 |
| 20/11/15 | M11 | 4.65 | 0 | 450 | 91.25 | 1.09 | WINTER | 444.62 | 36.78 | 31.73 | 0.86 | 4.88 | 179.33 | 12.51 | 12.51 |
| 18/11/15 | M12 | 4.7 | 0 | 450 | 91.07 | 1.29 | WINTER | 443.63 | 52.22 | 46.83 | 0.90 | 4.92 | 256.83 | 17.91 | 17.91 |
| 20/11/15 | M12 | 4.65 | 0 | 450 | 91.25 | 1.09 | WINTER | 444.62 | 32.56 | 34.61 | 1.06 | 5.12 | 166.77 | 11.63 | 11.63 |
| 22/11/15 | M12 | 4.65 | 0 | 450 | 92.65 | 0.9 | WINTER | 445.63 | 37.70 | 41.90 | 1.11 | 5.18 | 195.35 | 13.62 | 13.62 |
| 25/11/13 | M3 | 4.65 | 0 | 450 | 94.68 | 0.87 | WINTER | 445.87 | 33.17 | 36.28 | 1.09 | 5.16 | 171.18 | 11.94 | 11.94 |
| 2/12/13 | M3 | 4.6 | 0 | 450 | 92.02 | 0.93 | WINTER | 445.45 | 38.99 | 38.15 | 0.98 | 5.02 | 195.68 | 13.65 | 13.65 |
| 4/12/13 | M3 | 4.5 | 0 | 450 | 93.76 | 0.88 | WINTER | 445.78 | 69.31 | 72.94 | 1.05 | 5.11 | 354.14 | 24.70 | 24.70 |
| 6/12/13 | M3 | 4.5 | 0 | 450 | 93.57 | 0.86 | WINTER | 445.86 | 29.52 | 24.13 | 0.82 | 4.82 | 142.29 | 9.92 | 9.92 |
| 9/12/13 | M3 | 4.5 | 0 | 450 | 93.38 | 1.09 | WINTER | 444.75 | 29.38 | 24.89 | 0.85 | 4.86 | 142.71 | 9.95 | 9.95 |
| 15/11/13 | M4 | 4.55 | 0 | 450 | 94.1 | 1.05 | WINTER | 444.98 | 57.39 | 54.49 | 0.95 | 4.98 | 285.97 | 19.94 | 19.94 |
| 21/11/13 | M4 | 4.55 | 0 | 450 | 92.09 | 0.91 | WINTER | 445.55 | 45.27 | 32.06 | 0.71 | 4.69 | 212.16 | 14.79 | 14.79 |
| 25/11/13 | M4 | 4.65 | 0 | 450 | 94.7 | 0.86 | WINTER | 445.91 | 39.23 | 33.15 | 0.84 | 4.85 | 190.45 | 13.28 | 13.28 |
| 3/12/13 | M4 | 4.4 | 0 | 450 | 93.59 | 0.9 | WINTER | 445.67 | 39.68 | 35.38 | 0.89 | 4.91 | 194.92 | 13.59 | 13.59 |
| 5/12/13 | M4 | 4.45 | 0 | 450 | 92.51 | 0.84 | WINTER | 445.91 | 47.58 | 44.00 | 0.92 | 4.95 | 235.66 | 16.43 | 16.43 |
| 9/12/13 | M4 | 4.3 | 0 | 450 | 93.39 | 1.12 | WINTER | 444.60 | 36.02 | 31.83 | 0.88 | 4.90 | 176.59 | 12.31 | 12.31 |
| 19/11/13 | M5 | 4.5 | 0 | 450 | 92.53 | 0.92 | WINTER | 445.53 | 65.77 | 51.96 | 0.79 | 4.79 | 314.85 | 21.96 | 21.96 |
| 21/11/13 | M5 | 4.4 | 0 | 450 | 92.3 | 0.93 | WINTER | 445.47 | 37.10 | 30.99 | 0.84 | 4.84 | 179.68 | 12.53 | 12.53 |
| 3/12/13 | M5 | 4.4 | 0 | 450 | 93.54 | 0.9 | WINTER | 445.67 | 31.82 | 27.97 | 0.88 | 4.90 | 155.81 | 10.86 | 10.86 |
| 3/12/13 | M6 | 4.9 | 0 | 450 | 93.64 | 0.88 | WINTER | 445.77 | 41.78 | 31.82 | 0.76 | 4.75 | 198.56 | 13.85 | 13.85 |
| 9/12/13 | M6 | 4.8 | 0 | 450 | 93.42 | 1.09 | WINTER | 444.75 | 52.04 | 57.00 | 1.10 | 5.16 | 268.61 | 18.73 | 18.73 |
| 16/11/15 | M8 | 4.9 | 0 | 450 | 91.59 | 0.95 | WINTER | 445.33 | 49.57 | 44.11 | 0.89 | 4.91 | 243.37 | 16.97 | 16.97 |
| 18/11/15 | M8 | 4.85 | 0 | 450 | 91.03 | 1.22 | WINTER | 443.97 | 32.55 | 30.52 | 0.94 | 4.97 | 161.71 | 11.28 | 11.28 |
| 20/11/15 | M8 | 4.7 | 0 | 450 | 91.21 | 1.08 | WINTER | 444.67 | 34.64 | 24.56 | 0.71 | 4.69 | 162.39 | 11.32 | 11.32 |
| 18/11/13 | M1 | 4.2 | 0.5 | 450 | 91.85 | 1.05 | WINTER | 444.86 | 109.07 | 86.39 | 0.79 | 4.79 | 522.40 | 36.43 | 22.35 |
| 6/12/13 | M1 | 3.9 | 0.5 | 450 | 93.55 | 0.85 | WINTER | 445.91 | 86.85 | 68.51 | 0.79 | 4.79 | 415.65 | 28.98 | 17.19 |
| 16/11/15 | M10 | 4.65 | 0.5 | 450 | 91.64 | 0.98 | WINTER | 445.19 | 75.36 | 74.03 | 0.98 | 5.02 | 378.54 | 26.40 | 14.20 |
| 20/11/15 | M10 | 4.6 | 0.5 | 450 | 91.37 | 1.1 | WINTER | 444.58 | 91.44 | 82.19 | 0.90 | 4.92 | 449.94 | 31.38 | 21.42 |
| 16/11/15 | M11 | 4.7 | 0.5 | 450 | 91.65 | 1 | WINTER | 445.09 | 100.49 | 105.40 | 1.05 | 5.10 | 513.00 | 35.77 | 24.30 |
| 18/11/15 | M11 | 4.75 | 0.5 | 450 | 91.08 | 1.29 | WINTER | 443.63 | 73.93 | 70.41 | 0.95 | 4.99 | 368.64 | 25.71 | 15.27 |
| 20/11/15 | M11 | 4.65 | 0.5 | 450 | 91.25 | 1.04 | WINTER | 444.87 | 63.94 | 64.78 | 1.01 | 5.06 | 323.62 | 22.57 | 10.06 |
| 18/11/15 | M12 | 4.7 | 0.5 | 450 | 91.06 | 1.29 | WINTER | 443.63 | 88.56 | 65.95 | 0.74 | 4.73 | 419.01 | 29.22 | 11.31 |
| 25/11/13 | M3 | 4.65 | 0.5 | 450 | 94.67 | 0.88 | WINTER | 445.82 | 63.50 | 73.55 | 1.16 | 5.24 | 332.71 | 23.20 | 11.26 |
| 9/12/13 | M3 | 4.5 | 0.5 | 450 | 93.38 | 1.12 | WINTER | 444.60 | 82.53 | 94.97 | 1.15 | 5.23 | 431.63 | 30.10 | 20.15 |
| 19/11/13 | M5 | 4.5 | 0.5 | 450 | 92.51 | 0.89 | WINTER | 445.67 | 101.21 | 92.46 | 0.91 | 4.94 | 499.85 | 34.86 | 12.90 |
| 16/11/15 | M8 | 4.9 | 0.5 | 450 | 91.59 | 0.97 | WINTER | 445.23 | 100.62 | 92.30 | 0.92 | 4.94 | 497.42 | 34.69 | 17.72 |
| 18/11/15 | M8 | 4.85 | 0.5 | 450 | 91.17 | 1.2 | WINTER | 444.08 | 89.14 | 77.92 | 0.87 | 4.89 | 435.94 | 30.40 | 19.12 |
| 4/12/13 | M1 | 4.2 | 0.64 | 450 | 93.75 | 0.9 | WINTER | 445.68 | 56.08 | 61.55 | 1.10 | 5.16 | 289.63 | 20.20 | 13.30 |
| 16/11/15 | M10 | 4.65 | 0.64 | 450 | 91.64 | 1.01 | WINTER | 445.04 | 84.79 | 83.49 | 0.98 | 5.03 | 426.15 | 29.72 | 17.52 |
| 20/11/15 | M10 | 4.6 | 0.64 | 450 | 91.37 | 1.11 | WINTER | 444.53 | 102.39 | 97.53 | 0.95 | 4.99 | 510.59 | 35.61 | 25.65 |
| 16/11/15 | M11 | 4.7 | 0.64 | 450 | 91.65 | 1.01 | WINTER | 445.04 | 115.19 | 120.35 | 1.04 | 5.10 | 587.46 | 40.97 | 29.49 |
| 18/11/15 | M11 | 4.75 | 0.64 | 450 | 91.07 | 1.25 | WINTER | 443.82 | 116.61 | 103.48 | 0.89 | 4.91 | 572.16 | 39.90 | 29.47 |
| 18/11/15 | M12 | 4.7 | 0.64 | 450 | 91.07 | 1.29 | WINTER | 443.63 | 92.87 | 76.07 | 0.82 | 4.82 | 447.88 | 31.23 | 13.32 |
| 22/11/15 | M12 | 4.65 | 0.64 | 450 | 92.65 | 0.97 | WINTER | 445.29 | 72.30 | 73.44 | 1.02 | 5.06 | 366.18 | 25.53 | 11.91 |
| 3/12/13 | M4 | 4.4 | 0.64 | 450 | 93.6 | 0.93 | WINTER | 445.53 | 86.10 | 84.34 | 0.98 | 5.02 | 432.20 | 30.14 | 16.55 |
| 3/12/13 | M5 | 4.4 | 0.64 | 450 | 93.54 | 0.93 | WINTER | 445.53 | 72.90 | 75.86 | 1.04 | 5.09 | 371.41 | 25.90 | 15.03 |
| 16/11/15 | M8 | 4.9 | 0.64 | 450 | 91.6 | 0.98 | WINTER | 445.19 | 111.76 | 110.05 | 0.98 | 5.03 | 561.73 | 39.17 | 22.20 |
| 18/11/15 | M8 | 4.85 | 0.64 | 450 | 91.03 | 1.23 | WINTER | 443.92 | 74.24 | 66.31 | 0.89 | 4.91 | 364.82 | 25.44 | 14.16 |
| 18/11/13 | M1 | 4.2 | 0.75 | 450 | 91.86 | 1.08 | WINTER | 444.71 | 100.57 | 74.72 | 0.74 | 4.73 | 475.65 | 33.17 | 19.09 |
| 2/12/13 | M1 | 4 | 0.75 | 450 | 94.19 | 0.94 | WINTER | 445.51 | 70.93 | 67.61 | 0.95 | 4.99 | 353.74 | 24.67 | 10.89 |
| 16/11/15 | M10 | 4.65 | 0.75 | 450 | 91.64 | 1.05 | WINTER | 444.84 | 103.30 | 101.82 | 0.99 | 5.03 | 519.34 | 36.22 | 24.02 |
| 16/11/15 | M11 | 4.7 | 0.75 | 450 | 91.65 | 1 | WINTER | 445.09 | 138.69 | 146.63 | 1.06 | 5.12 | 709.44 | 49.47 | 38.00 |
| 18/11/15 | M11 | 4.75 | 0.75 | 450 | 91.04 | 1.35 | WINTER | 443.33 | 98.04 | 91.31 | 0.93 | 4.96 | 486.36 | 33.92 | 23.48 |
| 18/11/15 | M12 | 4.7 | 0.75 | 450 | 91.05 | 1.31 | WINTER | 443.53 | 105.61 | 97.28 | 0.92 | 4.95 | 522.57 | 36.44 | 18.53 |
| 22/11/15 | M12 | 4.65 | 0.75 | 450 | 92.65 | 0.94 | WINTER | 445.43 | 102.67 | 110.41 | 1.08 | 5.14 | 527.46 | 36.78 | 23.16 |
| 2/12/13 | M3 | 4.6 | 0.75 | 450 | 94.03 | 1.02 | WINTER | 445.12 | 125.52 | 91.94 | 0.73 | 4.72 | 592.03 | 41.28 | 27.64 |
| 21/11/13 | M4 | 4.55 | 0.75 | 450 | 92.06 | 0.92 | WINTER | 445.50 | 133.02 | 94.26 | 0.71 | 4.69 | 623.48 | 43.48 | 28.68 |
| 19/11/13 | M5 | 4.5 | 0.75 | 450 | 92.52 | 0.93 | WINTER | 445.48 | 103.80 | 101.10 | 0.97 | 5.01 | 520.34 | 36.29 | 14.33 |
| 16/11/15 | M8 | 4.9 | 0.75 | 450 | 91.6 | 0.98 | WINTER | 445.19 | 127.63 | 126.19 | 0.99 | 5.03 | 642.12 | 44.78 | 27.81 |
| 20/11/15 | M8 | 4.7 | 0.75 | 450 | 91.21 | 1.08 | WINTER | 444.67 | 127.21 | 118.82 | 0.93 | 4.96 | 631.45 | 44.03 | 32.71 |
| 16/11/15 | M10 | 4.65 | 0.88 | 450 | 91.63 | 1.02 | WINTER | 444.99 | 123.70 | 123.20 | 1.00 | 5.04 | 623.45 | 43.48 | 31.28 |
| 22/11/15 | M10 | 4.6 | 0.88 | 450 | 92.64 | 0.91 | WINTER | 445.58 | 129.98 | 132.94 | 1.02 | 5.07 | 659.39 | 45.98 | 34.58 |
| 16/11/15 | M11 | 4.7 | 0.88 | 450 | 91.65 | 1 | WINTER | 445.09 | 130.51 | 146.21 | 1.12 | 5.19 | 677.70 | 47.26 | 35.79 |
| 20/11/15 | M11 | 4.65 | 0.88 | 450 | 91.28 | 1.06 | WINTER | 444.77 | 107.85 | 111.24 | 1.03 | 5.08 | 548.28 | 38.23 | 25.73 |
| 20/11/15 | M12 | 4.65 | 0.88 | 450 | 91.22 | 1.08 | WINTER | 444.67 | 126.69 | 142.74 | 1.13 | 5.20 | 658.87 | 45.95 | 34.32 |
| 2/12/13 | M3 | 4.6 | 0.88 | 450 | 94.02 | 1.03 | WINTER | 445.07 | 120.49 | 90.26 | 0.75 | 4.74 | 570.73 | 39.80 | 26.15 |
| 4/12/13 | M3 | 4.5 | 0.88 | 450 | 93.76 | 0.9 | WINTER | 445.68 | 95.38 | 99.08 | 1.04 | 5.09 | 485.72 | 33.87 | 9.18 |
| 16/11/15 | M8 | 4.9 | 0.88 | 450 | 91.63 | 1.02 | WINTER | 444.99 | 164.21 | 180.01 | 1.10 | 5.16 | 847.85 | 59.12 | 42.15 |
| 18/11/15 | M8 | 4.85 | 0.88 | 450 | 91.11 | 1.23 | WINTER | 443.92 | 150.98 | 151.94 | 1.01 | 5.05 | 762.89 | 53.20 | 41.92 |
| 18/11/13 | M1 | 4.2 | 1 | 450 | 91.85 | 1.08 | WINTER | 444.71 | 118.52 | 92.06 | 0.78 | 4.77 | 565.45 | 39.43 | 25.35 |
| 6/12/13 | M1 | 3.9 | 1 | 450 | 93.54 | 0.88 | WINTER | 445.77 | 134.26 | 118.68 | 0.88 | 4.90 | 658.23 | 45.90 | 34.11 |
| 20/11/15 | M10 | 4.6 | 1 | 450 | 91.4 | 1.08 | WINTER | 444.68 | 161.46 | 176.32 | 1.09 | 5.16 | 832.81 | 58.07 | 48.12 |
| 18/11/15 | M11 | 4.75 | 1 | 450 | 91.12 | 1.28 | WINTER | 443.68 | 158.78 | 165.87 | 1.04 | 5.10 | 809.74 | 56.47 | 46.03 |
| 20/11/15 | M11 | 4.65 | 1 | 450 | 91.25 | 1.09 | WINTER | 444.62 | 141.63 | 146.02 | 1.03 | 5.08 | 719.91 | 50.20 | 37.70 |
| 18/11/15 | M12 | 4.7 | 1 | 450 | 91.05 | 1.32 | WINTER | 443.48 | 144.53 | 153.46 | 1.06 | 5.12 | 740.14 | 51.61 | 33.70 |
| 20/11/15 | M12 | 4.65 | 1 | 450 | 91.2 | 1.07 | WINTER | 444.72 | 143.04 | 164.59 | 1.15 | 5.23 | 748.10 | 52.17 | 40.54 |
| 2/12/13 | M3 | 4.6 | 1 | 450 | 94.03 | 0.97 | WINTER | 445.36 | 150.61 | 131.12 | 0.87 | 4.89 | 735.90 | 51.32 | 37.67 |
| 21/11/13 | M4 | 4.55 | 1 | 450 | 92.08 | 0.93 | WINTER | 445.46 | 162.33 | 137.70 | 0.85 | 4.86 | 788.72 | 55.00 | 40.21 |
| 18/11/15 | M8 | 4.85 | 1 | 450 | 91.07 | 1.25 | WINTER | 443.82 | 131.01 | 138.40 | 1.06 | 5.11 | 670.01 | 46.72 | 35.45 |
| 20/11/15 | M8 | 4.7 | 1 | 450 | 9.22 | 1.09 | WINTER | 396.80 | 121.91 | 130.10 | 1.07 | 5.13 | 625.07 | 43.59 | 32.26 |
